# Mechanical Properties of Nanoporous Metallic Ultrathin Films: A Paradigmatic Case

**DOI:** 10.3390/nano11113116

**Published:** 2021-11-18

**Authors:** Giulio Benetti, Francesco Banfi, Emanuele Cavaliere, Luca Gavioli

**Affiliations:** 1 Medical Physics Unit, Azienda Ospedaliera Universitaria Integrata, P.le Stefani 1, 37126 Verona, Italy; giulio.benetti@aovr.veneto.it; 2 FemtoNanoOptics Group, Université de Lyon, CNRS, Université Claude Bernard Lyon 1, Institut Lumière Matière, F-69622 Villeurbanne, France; francesco.banfi@univ-lyon1.fr; 3 Interdisciplinary Laboratories for Advanced Materials Physics (i-LAMP), Dipartimento di Matematica e Fisica, Università Cattolica del Sacro Cuore, Via della Garzetta 46, 25121 Brescia, Italy; Emanuele.Cavaliere@unicatt.it

**Keywords:** mechanical properties, nanomechanics, metallic nanoparticles, ultrathin porous films, granular nanomaterials, sensors, flexible solar cells, molecular dynamics, picosecond photoacoustic, ellipsometry, mechanical modeling

## Abstract

Nanoporous ultrathin films, constituted by a slab less than 100 nm thick and a certain void volume fraction provided by nanopores, are emerging as a new class of systems with a wide range of possible applications, including electrochemistry, energy storage, gas sensing and supercapacitors. The film porosity and morphology strongly affect nanoporous films mechanical properties, the knowledge of which is fundamental for designing films for specific applications. To unveil the relationships among the morphology, structure and mechanical response, a comprehensive and non-destructive investigation of a model system was sought. In this review, we examined the paradigmatic case of a nanoporous, granular, metallic ultrathin film with comprehensive bottom-up and top-down approaches, both experimentals and theoreticals. The granular film was made of Ag nanoparticles deposited by gas-phase synthesis, thus providing a solvent-free and ultrapure nanoporous system at room temperature. The results, bearing generality beyond the specific model system, are discussed for several applications specific to the morphological and mechanical properties of the investigated films, including bendable electronics, membrane separation and nanofluidic sensing.

## 1. Introduction

Typical porous films are formed by a metallic network containing pores, with sizes ranging from a few nanometers to more than one micron, depending on the synthesis technique [1], and with a thickness of more than 100 nm. The nanostructured nature of these systems provides a unique combination of physical properties—in particular, low density, high specific surface area [2], non-ohmic electric transport [3,4], and high specific strength [5]—while retaining some characteristics of bulk metals [1,6]. This results in a wide range of potential applications where such materials may be employed, e.g., bendable electronics [7,8], surface-enhanced Raman scattering [9,10,11], catalysis [12,13] and sensing [13,14], optical metamaterials [6], and microbicidal coatings [15,16,17].

The translation of such potential into real applications makes the knowledge of the films mechanics of paramount importance [5]. So far, most investigations have been performed on nanoporous gold films synthesized by dealloying, with thicknesses in most cases exceeding 100 nm [1,14,18]. The proposed picture describes the mechanical behavior as a function of microstructural parameters, such as ligament size and network connectivity [19]. For instance, the dependence of the film hardness on the inner ligament diameter was explained as a constrained motion of dislocations inside the structure [20], while the structural topology of the network was indicated as the key factor in determining the film’s mechanical response [19]. Two major limitations of the porous film synthesis, either by electrochemical dealloying or by employing polymeric templates, stand in the minimum film’s height that can be obtained and the pore dimensions [1].

Recently, it has been shown that it is possible to obtain much thinner porous film by a gas-phase synthesis technique, which is based on the assembly of elemental building blocks, namely nanoparticles (NPs), on a substrate, thus constructing the film via a bottom-up method [21,22,23,24,25]. The peculiarities of the deposition method allow for obtaining solvent-free and ultrapure nanoporous films that can be controlled in height down to a few nanometers and, most importantly, avoiding the synthesis-related complications involved in other methods. Here, we address the mechanical properties of such films for the case of Ag NP constituents. Nanoporous ultrathin films have been fully characterized in terms of compositional, structural, morphological, and mechanical properties, allowing for the establishment of a comprehensive experimental and theoretical description of their mutual dependence. The advent of a variety of applications based on these films triggered extensive interest in the assessment of their mechanical properties [26]. Specifically, the mechanical nanometrology of such assembled nanoporous systems may be achieved with a comprehensive combination of top-down and bottom-up approaches [25], as no single technique has proved to be sufficient per se to achieve a thorough understanding. For the above-mentioned reasons, the Ag NP film constitutes a paradigmatic system and an ideal playground to gain an overall comprehension of nanogranular film mechanics in general, that is, beyond the specific case addressed here.

**Figure 1:**
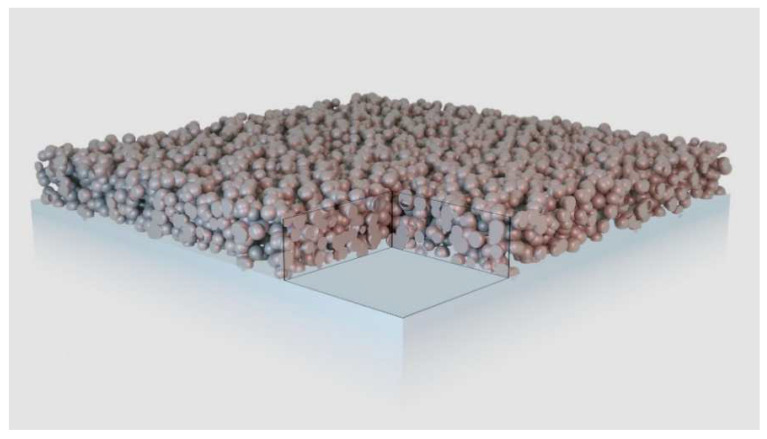
Schematic representation of an ultrathin porous film on a supporting substrate.

Figure 1 depicts an ultrathin metal porous film, a two-dimensional sheet of material with a typical thickness of 4 to 40 nm, composed of a metal network and a certain fraction of void. Very little is known about the mechanical behavior of this class of systems, as most of the evidence is sparse and based on a single-technique approach, unlike the case under discussion here. Being deposited on a support, the major application-oriented properties reside in the adhesion to the substrate and the mechanical resistance to wear, friction, and other agents during any deployment; the knowledge of the mechanical properties of porous metal systems are, thus, of paramount importance.

In this paper, we first introduce the open issues regarding the mechanical properties of porous films, starting with an account of the main techniques for the synthesis of such systems. We then summarize recent experimental and theoretical results obtained for the paradigmatic case of an ultrathin porous film—obtained by gas-phase deposition—in the context of the correlation between system morphology and mechanical properties. Finally, we discuss some applications based on the peculiar mechanical properties of the ultrathin film, such as selective gas membrane, nanofluidic sensor, and conductive transparent metal for flexible photovoltaic devices.

## 2. Open Issues in Ultrathin Nanoporous Films

### 2.1. Mechanical Properties

**Figure 2:**
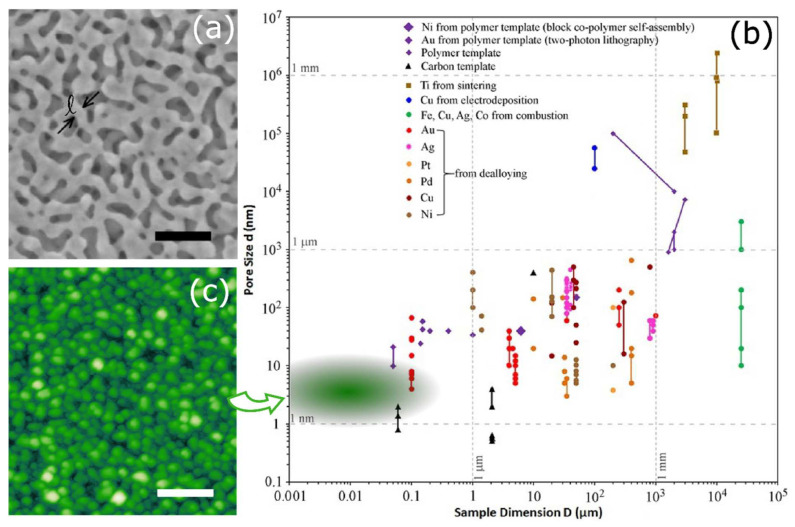
(**a**) Scanning electron micrograph of nanoporous gold with ligament size ℓ = 20 nm; scale bar = 100 nm (adapted from ref. [27], with permission from AIP Publishing). (**b**) Summary sample dimensions and pore sizes of nanoporous metal films fabricated by different synthesis methods. The green shaded area in the graph highlights the combination of possible pore sizes and film thickness obtainable by the gas-phase deposition discussed in this work. Adapted from ref. [1] under the Creative Commons Attribution License 4.0. (**c**) Representative AFM image of a nanogranular porous film at the same scale of panel (**a**). Scale bar = 100 nm, original data.

Before the advent of gas-phase deposition, a large portion of the current knowledge on the mechanical properties of nanoporous systems was obtained by investigating dealloyed Au thin films. Different techniques have been employed to measure the mechanical properties and disentangle the structure-properties relationship in such complex systems. The system is obtained by starting with an alloyed material, such as AuAg [13], and chemically removing the Ag, leaving a skeleton that forms the porous thin film (see Figure 2a) of the Au. The ligaments are the bridges connecting the larger grains of the skeleton, characterized by a size ℓ. The elastic modulus and yield strength may be obtained through micro-tension experiments that employ a complicated setup in which the elastic modulus is derived by depositing a film on a striped pre-patterned substrate and measuring the buckling of the stripes under compressive strain [27] or with uniaxial testing on millimeter-sized specimens [28]. The experimental results relate the different behaviors under tensile or compressive stress to the intrinsic structure of the film. In the former case, the failure of a small number of ligaments initiates a cascade of cell ruptures, leading to a macroscopically brittle specimen. Under compression, the structure locally densifies due to the presence of pores, leading to a very different yield strength [28]. This leads to a modified scaling exponent of the yield strength of Au NP that accounts for the influence of ligament size.

The role of network connectivity and microstructural parameters have also been studied employing scanning electron microscopy, positron lifetime, and Vickers hardness experiments, indicating that the hardness correlates with the ligament diameter with an exponent that is 40% lower than that of bulk gold [20]. Moreover, the influence of the topological structure of the porous network on the mechanical response was investigated by finite element simulations, showing that the macroscopic stiffness and strength are highly sensitive to the number of ligaments connected at a node [19]. The complicated effects to be taken into account to understand the mechanical properties also include the void size, the film thickness, and the applied load since they influence the surface energy and the corresponding surface stress at the nanovoids frontier [29]. Hence, the intrinsic structural arrangement and connectivity also impact nano-indentation measurements. The measurement of the stiffness and hardness of thin films and coatings [26,30] has to take into account the nano-indentation and plasticity effects [5], in particular, for Au nanoporous structures to provide meaningful results [31,32]. However, the typical indentation depths involved in the measurements, ranging from a few hundred nanometers to several microns [26], are larger than the ultrathin porous film thickness described in this review. This not only results in a major substrate contribution to the mechanical response but may also damage the film, thus posing fundamental limits to the reliability of nano-indentation measurements in such systems.

From the point of view of the techniques to measure the mechanical properties of ultrathin films, atomic force microscopy (AFM) is now widely used to obtain the stiffness and adhesion of materials at the nanoscale [33], and to extend the measurable dynamic range of the Young modulus [34]. However, even in bimodal tapping, the indentation depth may reach up to 10 nm, requiring the plastic deformations under load to be taken into account [26,33], and thus, hampering the determination of the mechanical properties of ultrathin porous films. A very recent study showed that AFM-modulated nano-indentation allows for the elasticity measurements of ultrathin materials due to the ability to keep the indentation depths lower than 0.1 nm. This novel method was applied to materials possessing a Young modulus in the 100–1000 GPa range [26]. An open issue remains for porous systems where mechanical deformations are determined not only by the bulk material but may be related to the presence of voids and dislocations where plasticity occurs [5]. For the sake of generality we note that for thicker films, ranging from hundreds of nanometers to the micrometer scale, the assessment of the mechanical properties may also benefit from other approaches [35,36].

An alternative route to measure the mechanical properties of thin films is the picosecond photoacoustic method, a non-invasive optical technique that relies on the excitation of the system’s mechanical breathing modes and their detection via the acousto-optic effect [37,38,39,40,41,42,43,44,45,46,47,48]. In brief, the energy delivered by a femtosecond laser pump pulse heats the film, triggering an impulsive thermal expansion. The oscillating strain field modulates the film’s dielectric constant, thus resulting in a time-periodic modulation of the sample’s optical transmission and reflectivity. We pinpoint that a typical displacement involved in picosecond photoacoustic experiments is in the range of 1 to 10 pm (depending on the exploited pump pulse energy) for thicknesses in the order of 10 nm, thus resulting in a strain in the order of 10^−3^–10^−4^. Hence, in its various forms, photoacoustic nanometrology is emerging as the go-to technique for the inspection of thin-film [39,49,50,51,52,53,54], ultrathin film [37,39,40,41,42,43], and nanoparticle [55,56] mechanics, and the topic is in rapid and continuous evolution. Cross-feed from other topical areas has recently resulted in novel inspection protocols based on the photoacoustic effects. For instance, advances in table-top UV laser technologies, coupled to state-of-the-art electron beam lithography, opened the way to the mechanics of thin films of thickness down to few nanometers [25,39,43].

### 2.2. Synthesis Routes and Pore Dimension

It is now accepted that, in a nanoporous thin film, the void size, *d*, and the system dimension, *D*, that is, the film thickness, are dependent on the synthesis process employed to fabricate the porous film [1,18,57]. The most common wet synthesis methods have been recently reviewed by Rebbecchi and Chen [18], including wet chemical etching (dealloying and electrodeposition) and polymer templating, while dry reactive ion etching has been summarized in the work of Tran et al. [57]. Although the known methods cover a wide range of pore sizes and sample thicknesses, many limitations are still faced with regard to controlling the pore size distribution, sample thickness, stability of the film, and composition [1,18]. From the plot reported in Figure 2b, one can appreciate the currently obtained pore size and sample dimension related to the different metals composing the porous film. As for dry etching processes for microfabrication technology, such as plasma etching, ion milling, sputter etching, and reactive ion etching, they are not yet applicable to obtain porous films below 100 nm [57]. In particular, it is worth noting that the lower limit of the thickness of the porous film (typically 100 nm) has been overcome only using a carbon template, and for Au, using a cumbersome lithographic process (see refs. In [1]). This leaves a wide field open for the development of ultrathin porous metal films, highlighted by the green shaded area in Figure 2b.

In this field, a few pioneering works have investigated the properties of porous films, through a gas-phase synthesis technique called supersonic cluster beam deposition (SCBD). The resulting systems typically present a granular morphology, as shown by the AFM image reported in Figure 2c. In particular, the surface roughness dependence and the porosity with respect to the growing film thickness have been measured for carbon [21,58,59], TiO_2_ [22,60,61,62,63], and Au [64], typically deducing the percentage of voids from measurements performed with a quartz microbalance. The results suggest an increasing porosity as a function of increasing film thickness, roughly placing this type of system in the shaded green area of Figure 2b. However, the actual knowledge of the pore size and distribution was never achieved due to the intrinsic difficulty of applying standard pore measurement techniques, such as the Brunauer—Emmett—Teller technique, to such solid thin systems. Extensive work remains to be done to define precise values of the pore size *d* vs. film thickness *D*. In a single case, through molecular dynamics simulations, a reconstruction of the film’s three-dimensional scaffold was obtained, thus providing some information on the pore distribution [25] and making the Ag case a unique example that is reviewed in detail in this work.

## 3. The Ag Ultrathin Porous Films

The Ag ultrathin porous films discussed in this review were obtained by direct deposition of Ag NPs from a supersonic beam, as detailed in [17,25,37,38], on different types of substrates as required by the specific experimental investigation involved. One of the key features is the reproducibility of the NP beam hitting the target substrate, which allows for the deposition and growth of the same film independently of the substrate type. This allowed us to tackle the understanding of the film’s physical behavior from the atomistic to the macroscopic description. The morphology, crystallinity, porosity, and void distribution at the nanometer scale, and the chemical composition, the oxidation state, and the optical response at the microscopic level are in fact required to properly understand the mechanical behavior of such systems. The theoretical and experimental investigation, merging bottom-up and top-down approaches, provided an explanation of the multifaceted topic of nanogranular material mechanics, a topic requiring a multi-technique approach [65,66].

### 3.1. Synthesis

The physical method employed to deposit assembled nanoparticle (NP) films is based on the principle of a spray can [59,67,68]. A supersonic beam of NPs is generated with the expansion of an NP gas mixture through a nozzle, previously generated by a high pressure (50 bar) spark plasma ablation of the desired material in a condensation chamber. The NPs are produced under a controlled environment and deposited in a medium vacuum, thus obtaining films of pure material on a large set of substrates, either rigid or flexible [7,15,69,70,71], without the presence of solvents or other contaminants, nor the need for post-annealing treatments that may alter the film morphology depending on the substrate used [72]. In view of the synthesis of porous films, a very important characteristic of this method is related to the average kinetic energy of the NPs (about 0.2 eV/atom) in the beam [68,73]. This translates into a soft landing that maintains the original NP structure and shape, thus giving rise to a porous film, as it has been shown for both metals and semiconductor materials [22,23,61,63]. The flexibility in the material composition and relative concentration demonstrated for this technique [24,74] makes it a valuable tool to grow and engineer porous films, overcoming the limitations encountered for the previously mentioned techniques.

### 3.2. Morphology

**Figure 3:**
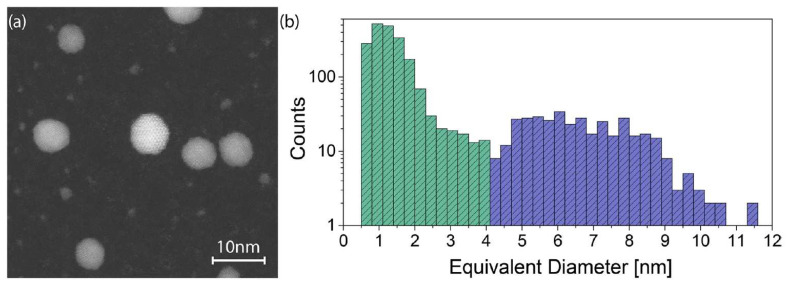
(**a**) HAADF-STEM image of scattered metallic Ag NPs deposited onto a carbon grid. (**b**) Size distribution in log-linear scale obtained from a collection of 100 STEM images of the as-deposited NPs. The distribution has been divided into two colored regions to highlight the partition into small (green) and large (blue) NPs. Reprinted with permission from ref. [25], further permission related to the material excerpted should be directed to the ACS.

One of the key factors in engineering an ultrathin film by assembling the nanoscale building blocks found in the supersonic beam is the characterization of such NPs. This is prodromic to the understanding of ultrathin film mechanics. SCBD allows for directly depositing the NPs onto transmission electron microscopy carbon grids at a low density, collecting NPs directly in flight, and thus avoiding possible diffusion-induced agglomeration and deformation of the NPs [23,25,68]. In Figure 3a, the high-angle annular dark field-scanning transmission electron microscopy (HAADF-STEM) image evidences the presence of two different types of NPs, the larger ones presenting a twinned crystalline structure, and the more numerous smaller ones with a size around 1 nm. A precise quantification of the NP size distribution, obtained from about one hundred images similar to that shown in Figure 3a, is plotted in the histogram of Figure 3b in a log-linear scale [25].

The bimodal shape of the distribution is evidenced by the green and blue colors for the small NPs (diameters up to 4.1 nm) and large NPs (diameters exceeding 4.1 nm), respectively. Considering the large number of images analyzed (around 100) and the fact that the particle shape and integrity are preserved at landing, since the kinetic energy per atom is significantly lower than the atomic cohesion energy of the atoms [25], the experimentally observed size distribution is very well representative of the NPs in the beam.

**Figure 4:**
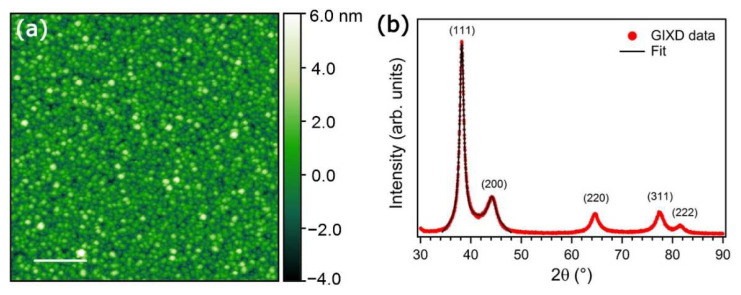
(**a**) AFM image of a 6-nm thick film deposited on a plain Si(100) wafer (original data). Scale bar = 200 nm. (**b**) Grazing-incidence X-ray diffraction taken on the Ag NP film. The reflections ascribed to polycrystalline Ag are reported together with the fit used to estimate the average grain size of the NPs. Adapted from the supporting information of [37] with permission.

Exposing a substrate to the supersonic beam for a given time gives rise to the assembly of the NPs into a film with a characteristic surface morphology; a typical nanogranularity is visible from the AFM image depicted in Figure 4a. The individual NPs are assembled into a uniform film with a surface roughness ranging between 1 and 2 nm, depending on the substrate on which it has been deposited [15,17,25]. Again, this characteristic property is directly correlated to the ballistic deposition regime provided by SCBD [68,73], which maintains the integrity of the single building block. A second important feature is the crystallinity of the NPs after landing on the substrate and assembling into a film. As shown in the graph of the grazing-incidence X-ray diffraction in Figure 4b, the prominent diffraction features belong to polycrystalline Ag [25], which is in agreement with the high-angle annular dark field scanning transmission electron microscopy (HAADF-STEM) observations. The profiles of the most intense reflections (i.e., (111) and (200)) were Gaussian fitted to estimate the coherency length from the Scherrer formula [75], providing a grain size of 6.5 ± 0.5 nm, in very good agreement with the distribution obtained from HAADF-STEM. Furthermore, the X-ray reflectivity data allowed us to determine the NP film density as ρ_NP_ = 8400 ± 600 kg/m^3^, just 0.8 times the bulk density of Ag [76].

**Figure 5:**
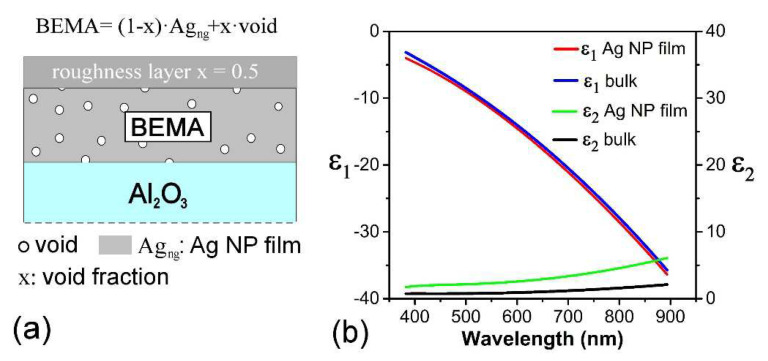
(**a**) Layer model scheme for nanogranular Ag NP film on Al_2_O_3_ substrate. The porosity of the film is introduced through the void factor (x) of the BEMA. Nano-granular Ag (Ag_ng_) is the host material. Surface roughness is considered through a roughness layer with a fixed void factor of x = 0.5. (**b**) Plot of the model optical dielectric functions ε_1_ and ε_2_, of the Ag NP film compared with reference data [80]. Adapted from [78] under the Creative Commons Attribution License 4.0.

The porosity of ultrathin nanogranular film deposited by SCBD can be obtained, in the first instance, by comparing the measured deposited mass from a quartz microbalance with the actual deposited thickness. A more refined method involves cross-analyzing Rutherford backscattering and X-ray photoemission spectroscopy (XPS) data [7,74]. This is achieved by comparing the number of atoms per unit area with the stoichiometry obtained by core level analysis. Another recently explored route, tested for the case of carbon NP-based ultrathin films, involves combining nitrogen adsorption isotherm analysis and AFM investigation [65,77]. The morphology and porosity of the nanogranular ultrathin film may also be extracted by appropriate modeling of spectroscopic ellipsometry data, as shown in [78]. Here, we detail the latter technique to some extent, as ellipsometry is among the most widely used thin film characterization techniques. Specifically, the film lying over the Al_2_O_3_ substrate is modeled as two layers, as sketched in Figure 5a, that are represented through the Bruggeman effective medium approximation (BEMA) [79]. The first layer is built as continuous bulk Ag, with voids circular in shape and a variable void fraction x. The further topmost BEMA layer is introduced with a fixed void factor of x = 0.5, to account for the surface roughness of the films.

The granular nature of the film is introduced with the free path effect correction Δε [81,82] due to size-dependent surface plasmon resonance on the bulk dielectric functions ε_b_ of silver metal, as follows:

(1) $$\varepsilon =\varepsilon_{b}+∆\varepsilon =\varepsilon_{b}+\frac{\omega_{p}^{2}}{\omega }\left[\frac{1}{\omega +i\Gamma_{b}}-\frac{1}{\omega +i\Gamma \left(R\right)}\right]$$

(2) $$\Gamma \left(R\right)=\Gamma_{b}+A\frac{v_{F}}{R}$$

where the plasma frequency of bulk silver is *ħω_p_* = 9.2 eV, and *Γ_b_* = 3.464 × 10^13^ s^−1^ is the scattering rate [83,84], *v_F_* = 1.4 10^6^ m/s [84] is the quasi-free electron velocity near the Fermi level, and *A* is a variable parameter related to the percolation of the NP film (*A* = 1 for continuous granular films [85]). The correction introduced via the *Γ* term connects the scattering time to *R*, the characteristic effective radius of the NP.

The dielectric function described in Equation (1) is used in the BEMA model to fit the ellipsometric data. The real and imaginary parts of the dielectric function (*ε*_1_ and *ε*_2_, respectively) obtained with the fitting model are shown in Figure 5b (red and green curves) and compared with the reference data used for bulk Ag [80] (black and blue curves). The roughness layer thickness of the model, 1.5 ± 0.2 nm, is in line with the actual film roughness (1.7 ± 0.2 nm) suggested by the AFM images [78], and the void factor of the BEMA layer (x = 17 ± 1) is compatible with the 20 ± 4% value from the X-ray reflectivity (XRR) measurements. Furthermore, from the fit value of *ħΓ(R)* = 0.13 eV, the extrapolated nanoparticle radius from the equation is *R* ≈ 7.5 nm, which is in very good agreement with the value estimated from the HAADF-STEM data. We highlight that the inclusion of the roughness layer, the finite-size effects (due to the NPs’ nanometric size), and the film porosity was already shown to be a fundamental asset in the modeling of the optical response of nanogranular Au films [86]. The fact that similar morphological features apply both to nanogranular Ag and Au films [86] supports the generality of the present results beyond the specific material.

### 3.3. Virtual Film Reconstruction

**Figure 6:**
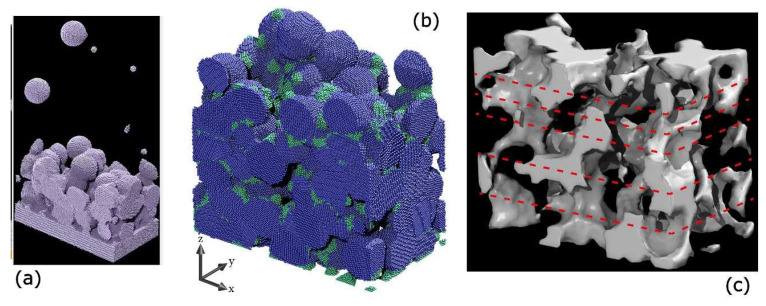
(**a**) MD simulation snapshot of the landing process employed to build the virtual film. (**b**) Rendering of the final virtual NP thin film. The Ag atoms composing big (set B) and small (set S) NPs are colored in blue and green, respectively, following the same color definitions as in Figure 3b. The average film thickness is 28.6 nm. (**c**) Void structure. The white 3D scaffold represents the voids between the deposited NPs. The cell base size for all panels is 35 × 20 nm^2^. Panels (**b**,**c**) are adapted from ref. [25], further permission related to the material excerpted should be directed to the ACS.

The experimental distribution of the NPs composing the nanogranular film is a key asset for devising simulations, yielding the so-called virtual film, that is, a calculated reconstruction of the nanogranular film scaffold. This is obtained via a bottom-up approach exploiting fully atomistic molecular dynamics (MD) simulations of the NPs’ landing processes [25] upon insertion of input parameters obtained from TEM images and gas dynamics data of the supersonic beams. The Ag−Ag interactions were represented with a 12-6 Lennard-Jones potential, while an embedded atom model potential (EAM) was adopted to estimate the film’s elastic constant [25]. To speed up computations, always accounting for the bimodal character of the experimental beam, the NPs sizes employed in the simulations were taken from the distribution shown in Figure 3b through a coarse-graining approach, downsizing the number of possible particle diameters to two, the small and big ones, with diameter *d_S_* = 1.6 nm and *d_L_* = 7.2 nm, respectively. The virtual NP film was obtained by simulating the Ag NPs to land in a sequential mode at 300 K to reproduce the actual growth process realized with SCBD. Figure 6a shows a time frame of this procedure, in which one can observe the growing film with the material already deposited on the substrate and some *d_S_* and *d_L_* NPs arriving in random locations of the simulation cell (base size of 35 × 20 nm^2^). Figure 6b clearly shows that the obtained film preserves the shape of the large NPs, shown in blue in Figure 6b, while the small NPs are indeed quite deformed, indicated in green. Since the small NPs account for just 5.4% of the total mass, they do not alter the overall granular nature of the film. The presence of connected pores can be appreciated in Figure 6c, as shown in a complementary image of panel (b). The resulting film porosity was calculated as *ϕ* = *V_p_*/*V* and reads *ϕ* = 0.27 ± 0.05, where *V_p_* is the void volume and *V* is the total NP film volume. The porosity of the film is a fundamental asset to rationalize the mechanical behavior of such systems, and the calculated value well matches the results obtained by quartz microbalance estimation and by XRR data [37]. Furthermore, the knowledge of the average pore size and distribution is useful when devising applications of films such as membranes.

### 3.4. Chemical State

**Figure 7:**
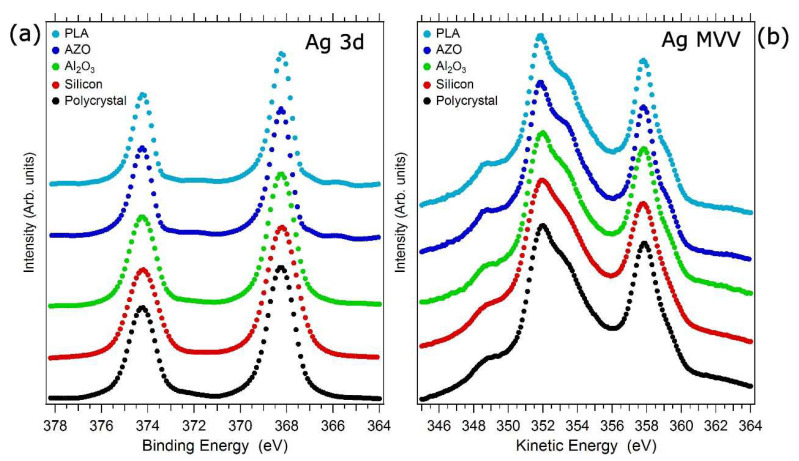
(**a**) XPS Ag 3d core levels and (**b**) Auger MVV Ag emission line obtained from Ag NP film deposited on different substrates. Ag polycrystal and Ag on Al_2_O_3_ taken with permission from ref [37]; further permission related to the material excerpted should be directed to the ACS. Silicon, aluminum zinc oxide (AZO), and polylactic acid (PLA), original data. The lines are reported after Shirley-type background subtraction and intensity normalization.

The SCBD synthesis technique produces a beam of NPs constituting, upon landing on a supporting substrate, a porous film. The question arises whether the Ag NPs, once the film is formed, form a metallic film independently on the substrate type. This is a key question, as the mechanics resulting from a metal or an oxide sample are potentially quite different. To answer this question, a set of X-ray photoemission spectroscopy (XPS) data, obtained from Ag NP films of 6 to 10 nm in thickness deposited on different substrates, are reported in Figure 7 for the Ag 3d core level and the Ag MVV Auger emission line. For a direct comparison with the behavior of a metallic system, we also reported the data taken on a sputtered-clean Ag polycrystalline substrate (black curves [37]). The main Ag peak binding energy in the different spectra (368.2 ± 0.1 eV) is identical, within the experimental resolution, to the measured metallic Ag reference (368.2 ± 0.1 eV). The data clearly indicate that the Ag 3d photoemission line is unaltered in energy position and shape independently on the substrate.

Similarly, the Auger MVV line shape of the Ag NP films is almost identical to the metallic Ag reference spectrum. The Auger parameter calculated from these data is 726.1 ± 0.1 eV for both the NP films and the metallic Ag reference, which is in very good agreement with the literature’s value for metallic Ag [87] and distinctly different from the AP of oxidized silver [88,89]. Therefore, the NPs forming the film grown by SCBD are composed of metallic Ag. Within the sensitivity of our XPS measurements, there was no evidence of spectral features related to Ag oxides independently from the used substrate.

### 3.5. Mechanical Properties

**Figure 8:**
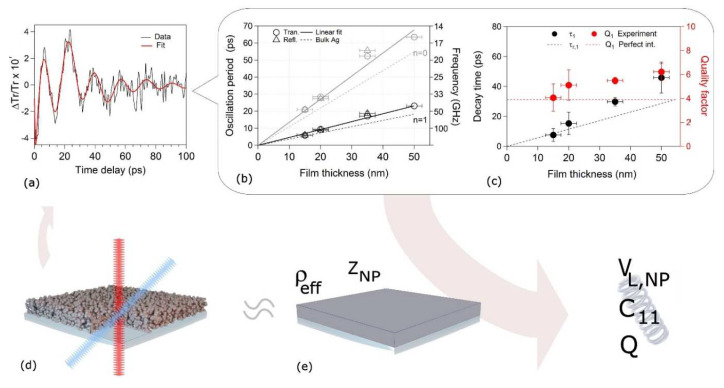
(**a**) Relative transmission variation vs. delay time acquired on a 35 nm thick NP film. Data (black line) and its fit are based on two damped oscillators (red line). (**b**) Oscillation period (left axis) and frequency (right axis) vs. film thickness obtained from transmission (circle) and reflection (triangle) geometry. Fundamental (*n* = 0) breathing mode data are in gray and first harmonic (*n* = 1) are in black. Full lines: linear fit through the origin of the data. Dashed lines: fundamental (*n* = 0) and first harmonic (*n* = 1) breathing mode period calculated from Equation (4), assuming bulk Ag values for the thin film. (**c**) Attenuation time (black, left axis) and quality factor (red, right axis) for the *n* = 1 breathing mode vs. film thickness. Markers: experimental data. Dashed line: theoretical values calculated from Equation (5) adopting the perfect interface model between the sapphire substrate and a homogeneous film with the same density and longitudinal sound velocity as those experimentally obtained for the Ag NP films, as exemplified in the scheme of panel (**e**). Adapted from ref. [37]; further permission related to the material excerpted should be directed to the ACS. (**d**) Scheme of the Ag NP film deposited on sapphire, evidencing the pump (blue) and probe (red) laser beams.

The mechanical properties of the Ag films deposited on a crystalline sapphire substrate were investigated by means of the picosecond photoacoustic technique [37,42,90]. In short, an ultrafast laser pump pulse heats the film’s lattice, triggering an impulsive thermal expansion of the film (see the scheme in Figure 8d), and ultimately exciting the system’s acoustic breathing modes. Consequently, the Ag film dielectric constant is modulated by the oscillating strain field, determining a time-periodic modulation of the film’s optical transmissivity and reflectivity. The delay time dependence of the relative transmission (Δ*Tr/Tr*), as measured via a time-delayed laser probe pulse, yields access to these modulations. To better understand the information extracted from this technique, it is worth examining some details of the experimental results.

Upon subtraction of a thermal background from the original optical signal [37], the mechanical response becomes evident in a delay time window ranging from a few picoseconds to 100 ps; see Figure 8a for a 35 nm thick film case. The data (black curve) are fit (red curve) by the sum of two exponentially damped oscillators in the following form:

(3) $$F_{n}\left(t\right)=A_{n}e^{-\frac{t}{\tau_{n}}}\cos\left(2\pi f_{n}+\varphi_{n}\right)$$

where *τ_n_*, *f_n_*, *φ_n_*, and *A_n_* are the n-th breathing mode decay time, frequency, phase, and amplitude contribution to the optical signal, respectively. The two damped oscillators (*n* = 0 and *n* = 1) have periods *T*_0_ and *T*_1_ and decay time constants *τ*_0_ and *τ*_1_, respectively. The oscillator periods, obtained experimentally in a similar way for different film thicknesses, are reported in Figure 8b for *T*_0_ (gray markers) and *T*_1_ (black markers), indicating a linear dependence on the film height. Since the *n* = 0 oscillator bears a quality factor of *Q*_0_ = π(*τ*_0_/*T*_0_) < 1, the oscillations last for a period or less. For this reason, only the *τ*_1_ (black circles) and *Q*_1_ (red circles) as a function of the film thickness are shown in Figure 8c [37].

In order to derive the film mechanical properties from the photoacoustic experiments, the data were rationalized by modeling the NP porous film with an isotropic and homogeneous layer, schematized in Figure 8e, possessing an effective density *ρ_NP_* and an effective stiffness tensor *C_NP_*. The model allows for calculating the breathing modes of the displacement field thickness (actually quasi-eigenmode) with the enforcement of the appropriate boundary conditions. The quasi-eigenmodes periods and lifetimes read as follows:

(4) $$T_{n}=h\;\frac{4}{v_{LNP}\left(1+2n\right)}$$

(5) $$\tau_{n}=h\;\frac{2}{v_{LNP}}{\left|ln\frac{Z_{S}-Z_{NP}}{Z_{S}+Z_{NP}}\right|}^{-1}\;\;$$

where *Z_i_* = *ρ_i_v_L,i_* is the acoustic impedance of material “*i*”, and stem imposing perfect contact boundary conditions, that is, continuity of stress and displacement fields, at the film-substrate interface.

Equation (4) was employed to fit the experimental data of Figure 8b with *v_L,NP_* as a free parameter, thus providing *v_L,NP_* = 2880 ± 40 m/s, the fitting curves of both breathing modes corresponding to the continuous lines. The obtained behavior was compared to the case of bulk polycrystalline Ag film, where *v_L,Ag_* = 3646 m/s [76], reported as dashed lines in Figure 8b, and proving the discrepancy between the Ag porous film and the polycrystalline Ag. The obtained wave velocity in the porous media is in fact 79% of the bulk counterpart.

Once the sound velocity is obtained from the fitting procedure, one exploits the assumption of a homogeneous and isotropic film (Figure 8e). In such a case, the material elastic properties are described by the two elastic stiffness tensor elements C_11,NP_ and C_44,NP_, where *C_11,NP_* = *ρ_NP_* (*v_L,NP_*)^2^. Here, the film density, obtained from different experimental measurements, was fundamental to properly determine the mechanical properties. By substituting the measured values, the results are *C_11,NP_* = 70 ± 5 GPa, half of that reported for bulk polycrystalline Ag, *C_11,Ag_* = 140 GPa [76], clearly showing that the nanoporous film was less stiff than the bulk Ag. Finally, the low residual stress, *σ_res_* = 70 ± 20 MPa, as deduced from X-ray reflectivity data [37], is in agreement with the trend obtained for nanoporous tungsten films grown by magnetron sputtering, where the residual stress reduction was obtained at a 30% porosity [91].

An alternative way to obtain the elastic constants of a granular/multi-element composite film relies on an effective medium approximation (EMA). Such an approach, widely employed to obtain the effective mechanical and optical properties of composite materials, is based on some assumptions and exploits the relative abundance and the properties of the different components of the heterogeneous material. For instance, through the formulation of Budiansky [92], it is possible to relate the effective bulk modulus K* and the effective shear modulus G* (also known as the second Lamé parameter or *C_44_*) of a porous material with the bulk *K* and *G* by means of the density only. In this case, the underlying assumptions of the model were (a) the spatial distribution of the different phases (e.g., silver and voids) inside the composite material must be homogeneous and isotropic, which means randomly positioned inclusions; (b) the composite material has to be formed with contiguous sphere-like grains, like in a polycrystal. Under the assumption of a homogeneous and isotropic composite material, *K* and *G* can be mapped to the other elastic moduli through the relations reported in Table 1. By employing the experimentally determined value of *ρ_NP_*, Budiansky’s EMA yields an effective value of C*_11_ = 66 GPa, a value very close to the experimentally observed one (70 ± 5 GPa), and a value of C*_44_ = 17 GPa. The C*_11_ and C*_44_ values completely characterize the elastic tensor of an effective homogeneous material. Furthermore, with the knowledge of the fundamental elastic tensor’s component, other mechanical properties of the NP film can be obtained, such as E*= 44.7 GPa and ν* = 0.327 [7].

An important point concerns the adhesion of the system to the substrate. Such information, which is hardly quantifiable with standard nano-indentation techniques or even by AFM, can be retrieved from the experimental film’s breathing mode lifetime τ and quality factor *Q* [41,50,51] as a function of the film thickness (via the fitting Equation (3)) that are plotted in Figure 8c as full black and red circles, respectively. In general, attenuation is due both to radiative and intrinsic losses, the latter occurring within the film’s bulk. However, it has been shown that the radiative channel is the leading mechanism ruling acoustic attenuation times for metallic thin films [41,51]. Equation (5) was used to determine the theoretical radiative attenuation time *τ*_*r*,1_ (dashed black line in Figure 8c) and, together with the period, to retrieve the theoretical quality factor *Q*_1_ (red dashed lines). The comparison to the reported data points indicates that such a model, which assumes a perfect film/substrate interface, indeed catches the main experimental features but underestimates both the attenuation times and the quality factors [37]. This behavior suggests that the nanoporous film is not perfectly adhered to the substrate, which is likely due to the fact that the granular and porous nature of the system causes the film/substrate interface area to have a patch-like arrangement, thus requiring a more realistic model.

**Figure 9:**
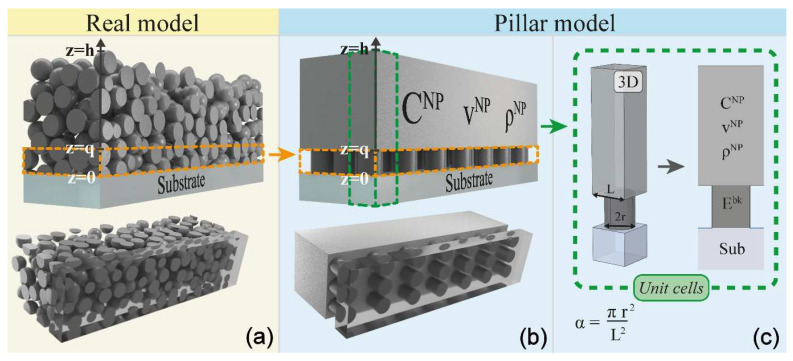
(**a**) 3D nanoparticle thin film of thickness h adhered on a semi-infinite substrate. The bottom view, as seen looking across the substrate, highlights the “patched” interface. (**b**) 3D pillar model: effective NP layer (*q* < *z* < *h*); pillars layer (0 < *z* < *q*); semi-infinite substrate (*z* < 0). The bottom view, as seen looking across the substrate, highlights the similarity with the “patched” interface of the real case. (**c**) Reduction of the periodic 3D pillar model to a single 3D unit cell of base size L*L. Adapted from ref. [93].

Recently, a 1D mechanical model for nanogranular films, based on a structural interface, has been presented to account for the patched interface [93]. The model, addressed as a pillar model, is based on a structural interface [94], meaning that a true structure is introduced to mimic the transition region between the NPs and the substrate. The pillar model splits the nanogranular film of thickness h (See Figure 9a) into three layers. The topmost layer is a homogeneous and isotropic thin film identical to the one previously presented (see Figure 8e), with effective mechanical properties obtained through Budiansky’s EMA. The intermediate layer (extending from the substrate to the homogeneous layer) is composed of pillars of height *q* and radius *r* and intends to mimic the mechanics in the interfacial layer—the patched interface between the actual EMA film and the bottom layer, that is, the substrate. The pillar’s density and Young modulus are taken as those of the real material of which the NPs are made—Ag. Hence, the pillar mechanical properties differ from those of the effective NP thin film layer since its longitudinal stiffness is dictated by the Ag Young modulus *E^Ag^* and not the effective C*_11_ (the pillars are free to expand transversely). The pillar model is more evolved with respect to spring-based interface models, which are commonly exploited to mimic imperfect interfaces [95]. In the present case, the pillar had rigidity *E^Ag^πr*^2^/*q*, which, contrary to the spring rigidity, arose from the specific interface geometrical and physical characteristics.

**Figure 10:**
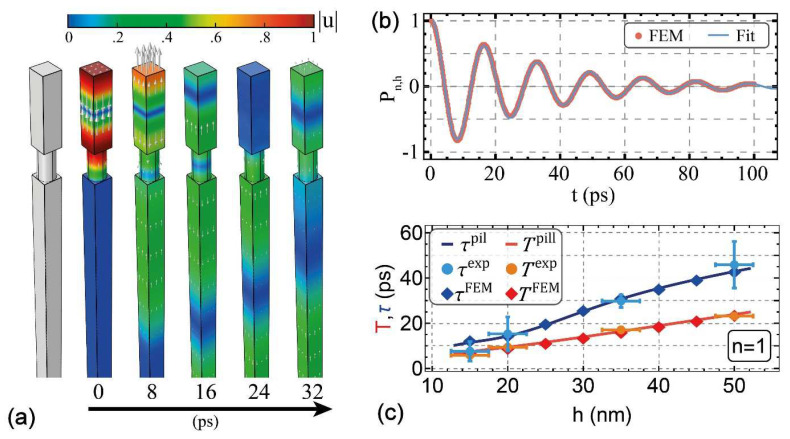
Pillar model FEM and analytical simulation results. (**a**) Simulation domain and displacement field (arrows) and modulus (color map) at increasing times for *n* = 1, *h* = 40 nm, and *q* = 12 nm. (**b**) Normalized projection coefficient *P_n_*_=1,*h*=40_ (i.e., the autocorrelation of the displacement field for the second eigenmode in a film with a thickness of 40 nm) vs. time for the case represented in panel (**a**) (full red dots); its fit with a damped oscillation of period *T* and decay time *τ* (blue line). (**c**) Periods and decay times vs. film thickness: FEM simulations (diamonds) and analytical 1D pillar model (solid lines) adapted from ref. [93] and experimental data (dots), adapted from ref. [37]; further permission related to the material excerpted should be directed to the ACS.

Furthermore, the pillars correctly account for inertia, as the mass is distributed as opposed to concentrated, as is the case for mass-spring interface models and alike. Tuning the only free parameters of the layer of pillars, namely the pillar height q and the pillar density α, it was possible to fit the experimental data, providing a remarkably accurate reproduction of both the experimental periods and decay times, reported in Figure 10c. The analytical solutions, obtained from the 1D pillar model, were also confirmed through time-dependent finite element method (FEM) simulations (Figure 10a,b). In this time-dependent simulation, the acoustic eigenmodes of the pillar configuration were computed and their frequencies and decay times were compared with the experimental data and analytical results from the pillar model (Figure 10c). The pillar model demonstrates that the mechanical mode lifetime, rather than its oscillation period, is most sensitive to the interface morphology, quantitatively explaining why, in previous photoacoustic experiments performed on granular thin films [37], the perfect adhesion model was able to correctly address the breathing mode oscillation period but failed in reproducing the lifetime.

**Figure 11:**
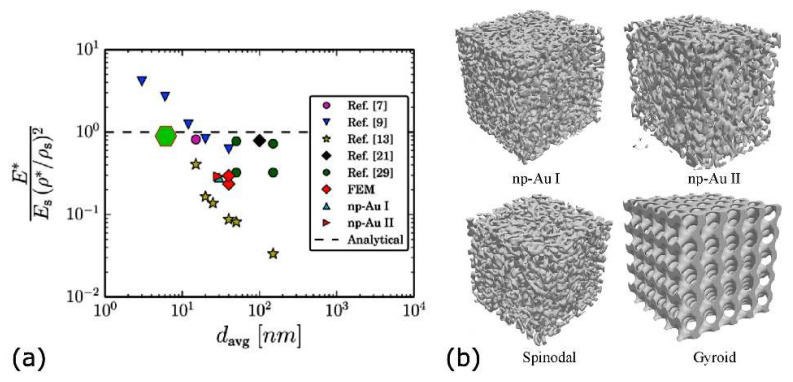
(**a**) Variation of normalized modulus of nanoporous gold with average ligament diameter. The dashed line represents the *C_E_* = 1 value. The currently discussed Ag case appears as the green hexagon. (**b**) Renderings of the reconstructed nanoporous gold structures, the computationally generated spinodal structure, and the mathematical gyroid structure. Adapted from ref. [19].

It is interesting to compare these results within the framework of a recent theoretical study in which the applicability of macroporous material elastic scaling laws to nanoporous foams was addressed [19]. The investigated foams consisted of nanoporous gold, which is considered a prototypical model material to study small-scale mechanical behavior. This system, exemplified in Figure 2a, is similar to the porous ultrathin film described here, although it is typically thicker and has a higher void percentage. In particular, the nanoporous gold was characterized by an irregular ligament geometry, presenting a local defect structure, which is believed to explain the trend of the increasing strength of the material for ligament sizes down to 10 nm. In the hypothesis that nanoporous Au behaves as an open-cell foam, the conventional macroscopic scaling law for macroporous cellular materials [96] takes the form of E*/*E* = *C_E_* (ρ*/*ρ_s_*)^2^, where E* and ρ* are the overall effective nanoporous system’s elastic modulus and density, and *E* and *ρ_s_* are the elastic modulus and density of the bulk solid. The adimensional prefactor *C_E_* is a function of the system geometry. The current debate indeed regards the applicability of such laws to Au foams [19,96,97], in particular for the assumption of *C_E_* = 1 to nanoscale Au porous films [19]. In the work of Mangipudi et al., a set of experimental measurements of the Young modulus was used to obtain *C_E_* as a function of the average ligament diameter *d_AVG_* for nanoporous gold (Figure 11a), and the results were discussed by proposing different topology and morphology models to calculate the film mechanical properties, reported in Figure 11b. Figure 11a shows a large variation (almost two orders of magnitude) of the *C_E_* prefactor with the ligament diameter, thus questioning the applicability of the macroscopic scaling laws for the case of nanoporous Au. The models allow for evaluating the effect of morphological factors (such as ligament non-uniform cross-sections, randomness, shape) and topological parameters (such as nodal connectivity, the number of “handles” in a connected structure) on the *C_E_*.

The results suggest that the macroscopic stiffness and strength, and hence, *C_E_*, are highly sensitive to the topology of the material (i.e., how the ligaments are organized in space) while being relatively less sensitive to the morphology. Thus, the work indicates that the large variations in the experimental data may be attributed to the experimental parameters of the synthesis methods, which are likely determining different topological arrangements of nominally similar foams.

The comparison of the present system with nanoporous Au was obtained by employing the Young modulus values previously obtained for the case of ultrathin nanoporous Ag film, and the estimation of *d_AVG_* = 7 nm from MD simulations, resulting in a value of *C_E_* = 0.97 ± 0.09, reported as a green hexagon in Figure 11a. The obtained value falls close to the *C_E_* = 1 assumption, which would extend the macroscopic scaling law for macroporous cellular materials below the limit experimentally obtained with nanoporous Au. This is a surprising result since, (a) for Ag, the scaffold obtained by MD simulations clearly showed that the ligaments contained grain boundaries derived from the assembly mechanism of the NPs, while in nanoporous gold, the ligaments originated from the dealloying process; (b) the void density of the Ag was 20% as compared to the values larger than 50% of the Au foams. This comparison indicates that the new class of nanoporous material, exemplified by the case under discussion here, may serve as a paradigmatic system for the structure-topology-mechanical properties investigation of the mechanical properties at the nanoscale typically accessed by nanotomography [98].

## 4. Applications

In this section, our review of several applications of the Ag ultrathin metal porous film is presented, expanding on their peculiar mechanical properties.

### 4.1. Gas Membrane

The inherent open-pore granularity, together with a pore’s dimension below the gas molecules mean free path, and the fact that gas-phase deposition techniques allow for depositing NP porous ultrathin film onto virtually any surface, makes the system appealing for distributed gas dynamics applications [25]. Knowledge of the pores connectivity pattern and the transport mechanism ruling the gas dynamics may both be obtained from the simulated virtual film. The pore structure is obtained as a complement of the NP distribution, as shown in Figure 6c. In order to determine which gas transport regime takes place across the ultrathin film at standard atmospheric conditions, the Knudsen number *K_n_ = λ/L* [25] needs to be inspected. In the latter formula, *λ* is the molecular mean free path and L is the average separation between two sides of the pipe (mimicking the scaffold’s open channel, i.e. voids) the molecules are traveling into, thus determining the scattering event between the gas molecule and the NP film scaffold. This value is estimated by calculating the average cylindrical cross-section of the voids, giving a radius of *L* = 5 nm, which is an overestimation of the effective *L* since the actual void cross-sections are not circular. For gases such as helium and chlorine, one finds that *K_n_* ≈ 10–30, that is, gas transport falls in the molecular regime. Such features make the NP film an appealing inorganic nanoporous membrane for distributed membrane-based gas separation [99].

In the future, the possibility of depositing such a controlled ultrathin porous films, working as membranes on suited supports, would allow for overcoming some of the inherent limitations of currently available polymer membranes [99]. In particular, depositing a porous film on a polymer membrane support would provide the possibility of exploiting the gas selectivity of the metal portion while conserving the permeability of the polymeric membrane. This could repose on the metal ultrathin layer’s thickness, which is one to two orders of magnitude smaller than that of the supporting polymer membrane. Furthermore, the larger resistance to bending of the NP film [7] would also help to maintain the elastic properties of the support membrane. This aspect is further detailed in Section 4.3.

### 4.2. Nanofluidic Sensing

**Figure 12:**
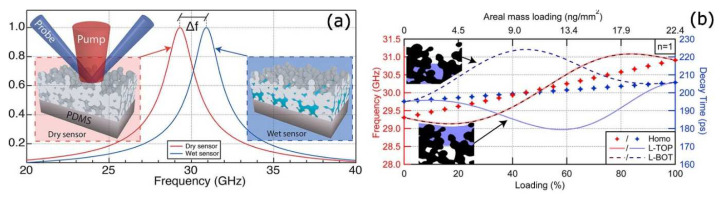
(**a**) Resonance behavior for the n=1 acoustic breathing mode measured by the photoacoustic signal expected for a dry (red) and fully infiltrated (blue) granular thin-film sensor. Upon water infiltration, the acoustic resonance undergoes a frequency shift of Δ*f* = *f_w_* − *f_d_*. The two insets represent the dry and fully infiltrated device, respectively, together with a schematic of the pump and probe technique. (**b**) Frequency of the *n* = 1 acoustic breathing modes (left axis, red) and decay times (right axis, blue) vs. water filling within the layered adsorption scenarios L-TOP (full lines), L-BOT (dashed lines), and, for the sake of comparison, for the homogeneous wetting case (markers). Water filling is expressed both as relative volumetric loading (bottom axis) and equivalent areal mass loading (top axis). Illustration: schematics of the infiltrated device for the L-TOP (bottom picture) and L-BOT (top picture) scenarios. Water is depicted in blue, and silver is depicted in black. Adapted from ref. [38]; further permission related to the material excerpted should be directed to the ACS.

Nanoporous films are emerging as favorable systems to be employed in nanofluidics [100,101], although the measurements of their permeability remain an issue due to the limited sensitivity and difficulty in the data analysis of environmental ellipsometric porosimetry and gas adsorption measurements. A possible alternative is based on the same optical picosecond photoacoustic measurements performed on the ultrathin porous films discussed in Section 3.4. The practical example for the case of the Ag porous film deposited on polydimethylsiloxane (PDMS) is depicted in the left inset of Figure 12a, although the reasoning can be extended to other types of materials. PDMS is a suitable support since it yields a high acoustic film-substrate mismatch, thus increasing the film’s mechanical breathing mode lifetime and quality factor.

To describe the sensing behavior of the nanoporous film under illumination by a short laser pulse, one has to start from the contribution to the optical signal of the *n* = 1 film breathing mode described by Equation (3). Then, by calculating the Fourier transform of the time-resolved trace, one obtains a resonance line shape of the following form:

(6) $$\left|\tilde{F}\left(f\right)\right|=\left|A\right|\frac{\tau }{2\sqrt{1+{\left(2\pi \tau \left(\left|f\right|-f_{d,w}\right)\right)}^{2}}}$$

where *A* and τ are the amplitude and decay time of the breathing mode, respectively, and *f_d,w_* indicates the resonant frequency of the breathing mode for the case of a dry (*d*) or wet (*w*) film, respectively. The curve of the dry device response (i.e., non-infiltrated by water) is provided as the red curve in Figure 12a. Upon fluid infiltration in the granular film, both the system’s density and inverse stiffness constant increase, thus leading to a different resonance frequency *f_w_*. The new device response gives rise to the blue curve of Figure 12a, corresponding to a resonance shift of 1.61 GHz [38]. The sensing principle of a device is based on the frequency shift measurement with respect to a calibrated response.

Apart from a generic fluid infiltration sensing scheme, a further development would be the ability to discriminate among different infiltration patterns, a yet unsolved issue in nanoporous materials [102,103]. As an exemplifying case, two possible layered infiltration patterns were analyzed. When the infiltrated water layer resides on the top portion of the film, likely due to limited pore accessibility [104], one has the layer on top (L-TOP) case, depicted in the inset at the bottom left corner of Figure 12b. In the opposite scenario (L-BOT), an infiltrated water layer rests on the polymeric substrate, as shown in the top left corner of Figure 12b. Such a configuration may derive from partial water evaporation from a fully infiltrated sensor. For both the L-TOP and L-BOT cases, the system was modeled as a two-layered effective medium adhering on the semi-infinite PDMS substrate. The L-TOP configuration consists of a fully infiltrated Ag scaffold (Ag and water-filled voids), followed by a dry Ag scaffold with voids. The layers’ order was inverted for the L-BOT case.

The mechanical breathing mode frequency and decay time for the *n* = 1 mode are reported in Figure 12b as a function of the film water’s relative loading. The striking feature is the non-linear response as compared to the homogeneous wetting case (diamonds), hence providing a way to distinguish the homogeneous from the non-homogeneous types of infiltration. The data of Figure 12b shows further that the L-TOP and L-BOT are characterized by the same frequency versus the loading curve (continuous and dashed red lines, respectively) since the nanoporous film-PDMS interface is substantially stress-free, and hence, the device behaves as a free-standing layered membrane, that is, symmetric for the L-TOP and LBOT cases.

Conversely, the decay time versus loading curves (continuous and dashed blue lines in Figure 12b) show opposite trends in the L-TOP and L-BOT configurations. For any quantity of adsorbed water, τ is the smallest for the L-TOP and the biggest for the L-BOT, and sits between the two for the homogeneous filling case. Such behavior is related to the acoustic impedance jumps across the film. The smoother the impedance changes across the device, the higher the acoustic wave transmission to the PDMS substrate is, and the lower the breathing mode decay time is.

### 4.3. Flexible Multilayers for Solar Cells

**Figure 13:**
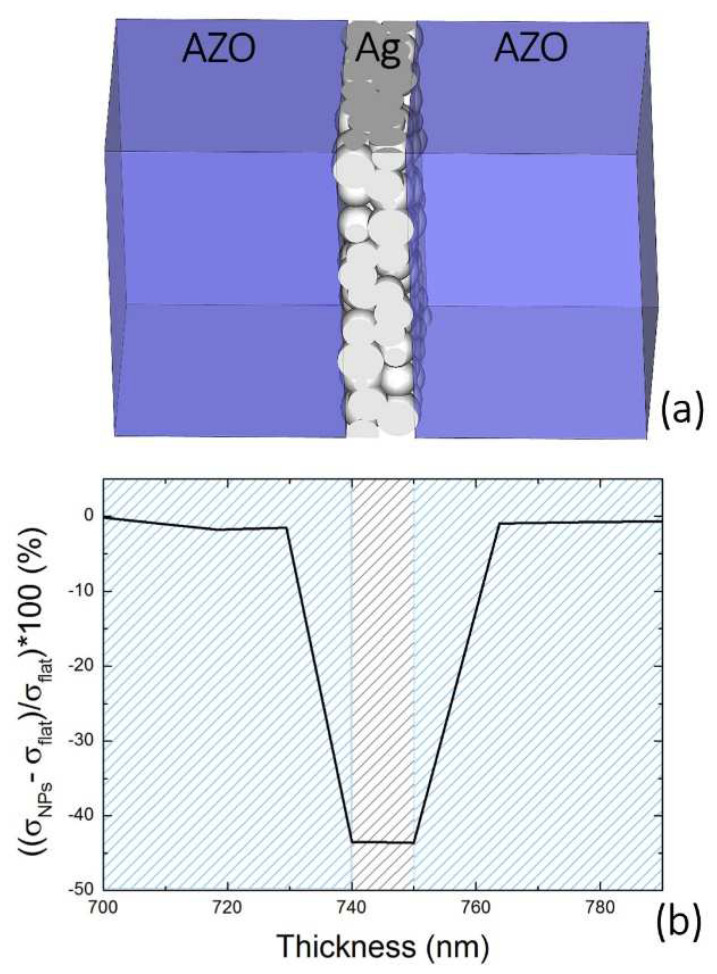
(**a**) Schematic of the sandwiched multilayer structure (AZO/Ag/AZO) described in the experiment. (**b**) Relative difference variation of the stress σ for the Ag sputtered film (*σ_flat_*) and the AgNPs (*σ_NPs_*) normalized against *σ_flat_* versus the multilayer depth. Adapted from ref. [7].

Flexible solar cells is another area in which the mechanical properties of ultrathin porous films can make a difference. The production of flexible solar cells requires the synthesis of new kinds of transparent conductive oxides (TCO) [7]. Specifically, new transparent and conducting materials (TCM) have been proposed to replace or reduce the expensive and toxic indium tin oxide [8,105,106]. A critical issue, however, remains with regard to the electrical resistance and mechanical wear of the device upon bending. In this sense, the scope is to develop a material combination, yielding an optimal balance among optical transparency, electrical conductivity, and mechanical resistance to wear upon bending. Several strategies have been pursued to this end [107,108,109,110], among them, the exploitation of metallic NP film sandwiched between two TCMs seems very promising. A recent study by Torrisi et al. evidenced the peculiar mechanical properties of the assembled nanogranular nature of Ag films in a sandwiched multilayer configuration for a flexible solar device [7], as schematized in Figure 13a. The system was obtained by stacking aluminum zinc oxide (AZO) and Ag layers on a transparent substrate in an AZO/Ag/AZO sequence 40/10/40 nm thick, respectively.

Two different Ag layers, one homogeneous (obtained by magnetron sputtering) and one nanogranular (obtained by SCBD), were investigated by optical transmission, electrical resistivity measurements, Rutherford backscattering (RBS), and SEM. The mechanical reliability under bending obtained from the experimental data was also theoretically investigated with a multiscale finite element simulation. RBS was employed to identify the film thickness and to ensure the reproducibility of the multilayer for the Ag films deposited with SCBD and RF magnetron sputtering. The obtained films present a thickness of 40 ± 4 (AZO) and 10 ± 1 (Ag) nm, showing that comparable systems were experimentally tested.

The nanogranular Ag layer outperformed the magnetron sputtered layer in terms of improved optical transmission and decreased reflectance in the visible region while maintaining the same electrical resistivity. Most importantly, the mechanics of the nanogranular film came into play when the effect on the relative stress variation inside the film was considered. Figure 13b shows the comparison of the simulated relative stress variation across the AZO/Ag/AZO sandwich thickness for the case of Ag sputtered film (*σ_flat_*) and for the case of Ag NP film (*σ_NPs_*). The plot clearly shows that there is a stress reduction of at least 40% at the AZO/Ag interface and in the core of the Ag nanogranular film with respect to the magnetron sputtered layer. This is also reflected in the SEM investigation of the multilayers subjected to bending. The bending-induced fracture in the multilayer containing the magnetron sputtered Ag film is at least four times larger than that of the multilayer containing the Ag NPs. Furthermore, the surface morphology of this latter sandwich structure is definitely smoother. Such behavior can be explained considering that the in-plane elastic stiffness *C*_44_ of the NP film layer was inferior to that of polycrystalline Ag, thus reducing the shear forces upon bending as compared to a homogeneous Ag film. Hence, the Ag NP film should better relax the augmented stress introduced during a bending process by accommodating the NPs in a way similar to sliding beads in a bending necklace, thus justifying the augmented flexibility and mechanical reliability observed with multiple bendings.

## 5. Conclusions

We reviewed the mechanical properties of a Ag ultrathin porous metallic film. This system emerged as a paradigmatic case of a nanoporous system for the four following reasons: (1) the peculiarities of the deposition method allowed us to obtain solvent-free and ultrapure nanoporous films made of juxtaposed NPs, the height of which can be controlled to few nanometers and, most importantly, avoiding the synthesis-related complications involved in other methods; (2) it has been investigated in detail, both with top-down and bottom-up approaches, exploiting a variety of techniques, and hence, allowing us to correlate the morphology with the mechanical response (3) the NP material and substrate may be varied almost at will; (4) several applications, based on the mechanical properties of this system, have recently been proposed. Specifically, we summarized the experimental results and the models developed to investigate and benchmark the morphological, compositional, and mechanical acoustic properties of such films. The film characteristics, that is, morphology, porosity, chemical state, composition, morphology-related optical constants, Young modulus, and residual stress, were experimentally obtained through the bottom-up and top-down investigations exploiting HAADF-STEM, AFM, X-ray diffraction analysis, Rutherford backscattering analysis, spectroscopic ellipsometry, XPS, and time-resolved optical pump-probe spectroscopy. Furthermore, we reviewed the results obtained from different models employed to analyze and interpret the experimental data. MD simulations provided a virtual film scaffold, allowing us to retrieve morphological and mechanical properties. The effective medium approximation was used to describe the morphology starting with the optical constants of the nanogranular film, while a fully analytical model allowed us to unveil the role of the inner film morphology in determining the mechanical acoustic response of the system.

Furthermore, we reviewed recently proposed applications based on the mechanical properties of the ultrathin nanogranular films—gas membrane separation based on the nanometric pore sizes, nanofluidic sensing determined by the film’s acoustic response, and outstanding optical transmission coupled with electrical conductivity and mechanical resistance to bending in flexible photovoltaic devices. This review also evidenced that nanoporous metallic films and coatings, synthesized by the direct deposition of NPs, are extending the accessible mechanical and morphological properties beyond the limits currently investigated in porous systems obtained by wet chemistry or dry etching. Gas-phase synthesis (e.g., SCBD), indeed, provides the flexibility to access ultra-thin film with porosities spanning from 0.3 to 0.5 and thicknesses in the order of tens of nanometers, a range for which the literature on the chemically synthesized NP film is scarce.

In the future, the investigation may be expanded to include the nanomechanics inherent to nanoporous thin films composed of multiple elemental NPs, such as metal and oxides, and varying the filling fraction of the films. Knowledge of the mechanical response of such systems would open numerous applications, such as resistive switches, all-optical switches based on the absorption characteristics of ultrathin bi-elemental films, and wide-spectrum antibacterial or optical applications, just to mention a few.

## Figures and Tables

**Table 1:** Relations between different elastic moduli for homogeneous and isotropic materials. *E* is the Young modulus, *ν* is the Poisson’s ratio, *C*_11_ is the first matrix element of the stiffness tensor, and *λ* is the first Lamé parameter. The parentheses indicate that the quantity is a function of two variables.

	K(x,x)	G(x,x)	E(x,x)	ν(x,x)	$C_{11}\left(x,x\right)$	λ(x,x)
(K,G)	K	G	$\frac{9KG}{3K+G}$	$\frac{3K-2G}{2\left(3K+G\right)}$	$K+\frac{4G}{3}$	$K-\frac{2G}{3}$
(E,ν)	$\frac{E}{3\left(1-2\nu \right)}$	$\frac{E}{2\left(1+\nu \right)}$	E	ν	$\frac{E\left(1-\nu \right)}{\left(1+\nu \right)\left(1-2\nu \right)}$	$\frac{E\nu }{\left(1+\nu \right)\left(1-2\nu \right)}$
$\left(\lambda ,C_{11}\right)$	$\frac{C_{11}+2\lambda }{3}$	$\frac{C_{11}-\lambda }{2}$	$\frac{\left(C_{11}-\lambda \right)\left(C_{11}+2\lambda \right)}{C_{11}+\lambda }$	$\frac{\lambda }{C_{11}+\lambda }$	$C_{11}$	λ
